# Blood speckle tracking to predict functional status in pediatric patients with dilated cardiomyopathy

**DOI:** 10.1186/s12872-025-04984-2

**Published:** 2025-07-21

**Authors:** Antoine Fakhry AbdelMassih, Soha Emam, Aliaa Ibrahim Mabrouk, Manal AbdelHameed

**Affiliations:** 1 Department of Pediatrics, Pediatric Cardiology Division, Faculty of Medicine, Specialized Pediatric Hospital, Cairo University, Cairo University Children Hospital, Kasr Al Ainy Street, Cairo, 12411 Egypt; 2 Pediatrics’ Master of Science training program, Faculty of Medicine, Cairo University Children Hospital, Cairo University, Cairo, Egypt

**Keywords:** DCM, NYHA, Vortex timing, Post-systolic index, Dyssynchrony

## Abstract

**Background:**

Dilated Cardiomyopathy (DCM) is an important cause of significant cardiac functional impairment in pediatric patients. There is to date debate on which echocardiographic variable correlates better with the functional status of the patient.

**Methodology:**

28 DCM patients (NYHA class II and III), and 30 controls were enrolled in the study, where advanced echocardiographic examination was used to assess LV (Left ventricular) function. Tissue Doppler, 2D speckle tracking for PSI (post-systolic strain index calculation), and blood speckle tracking for the timing of LV vortex formation were implemented.

**Results:**

Intriguingly, none of LV GLS and GAS (Global longitudinal and area strain), or EF (Ejection fraction, were able to differentiate NYHA II and III subgroups of cases. In contrast, PSI, and systolic vortex formation were significantly different between subgroups of cases; systolic vortex was seen in 33% of NYHA II patients compared to 77% of NYHA III patients, a PSI median value of 5% was seen in NYHA III cases, and a cut-off >4% was 92% sensitive in predicting worse manifestations.

**Conclusion:**

Dyssynchrony; seems to play a significant role in orchestrating symptoms of heart failure. In this context, blood speckle tracking study seems a promising bedside method, in its assessment. A large room for improvement and systemization of its assessment is still needed to objectivize its use.

**Supplementary Information:**

The online version contains supplementary material available at 10.1186/s12872-025-04984-2.

## Background

Dilated cardiomyopathy is a very heterogeneous disease, its heterogeneity does not lie only in its etiology but also in its symptoms and outcomes [1]. 

The heterogeneity of symptoms and outcomes in DCM has been attributed to genetic heterogeneity, a study by Kayvanpour and colleagues, demonstrated that some mutations are linked to the severity of manifestations, namely PLN and LMNA mutations [2]. 

Another study by Halliday et al. demonstrated that the extent of gadolinium enhancement is an important predictor of NYHA classes and adverse events, the same research shows that the Ejection fraction was 40% in patients with no late gadolinium enhancement (LGE), despite the heterogeneity of manifestations within this group, as 55% of patients were NYHA II-IV compared to a 45% of patients within the NYHA I category [3]. 

There is, unfortunately, no single study that investigated the severity of heart failure manifestations, in relation to functional echocardiographic parameters.

Moreover, left ventricular dyssynchrony, especially intraventricular dyssynchrony is thought to decrease pump efficiency by disorganizing cardiac contraction [4].

**Fig. 1:**
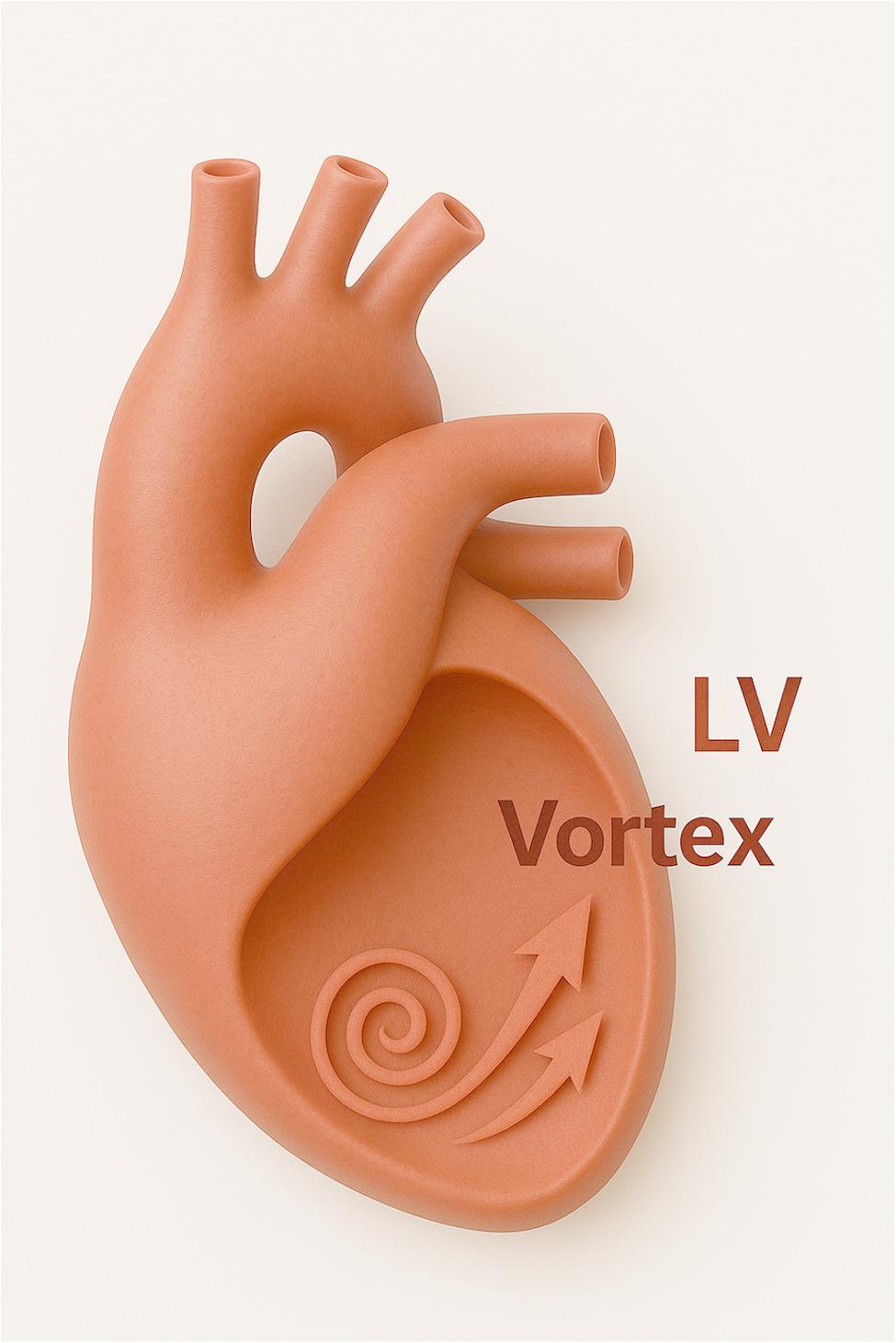
Schematic diagram illustrating LV vortex formation. Abbreviations: LV: left ventricle

A left ventricular vortex refers to an organized, rotational flow pattern of blood that develops within the ventricular cavity during diastole. This vortex formation results from the complex interplay of myocardial motion and blood inertia, facilitating efficient blood filling and ejection by promoting smooth blood flow dynamics. The presence and characteristics of the vortex can serve as indicators of ventricular systolic and diastolic function, reflecting the efficiency of intracardiac flow patterns (Fig. 1). The timing of vortex formation inside the left ventricle is slowly becoming an area of study of different cardiovascular disorders and is hypothesized to affect cardiac output. Their quantitative assessment has been made available, via non-invasive techniques, such as cardiac magnetic resonance imaging (CMR) and some echocardiography-based software [5]. 

The role of Cardiac Magnetic Resonance (CMR) imaging in the initial classification of Dilated Cardiomyopathy (DCM) is significant, as it offers detailed insights into myocardial structure and function. However, its routine use for follow-up assessments, particularly in pediatric populations and developing countries, is limited due to factors such as high costs, limited access to CMR equipment, and the need for general anesthesia in children, which complicates the process. Consequently, echocardiography often remains the primary imaging modality for follow-up due to its greater availability, lower cost, and ease of use, despite its limitations compared to CMR. To improve patient management in these settings, targeted guidelines on CMR usage for follow-up and the incorporation of alternative imaging technologies, along with ongoing education and training, may enhance the quality of care for DCM patients.

For this reason and since CMR is not routinely done in our center for follow up of DCM patients, we aimed in this study, to explore different advanced echocardiographic variables in relation with NYHA class of heart failure, in order to determine which parameter can accurately predict worse manifestations.

## Methodology

### Study subjects and setting

This study was designed as a case-control study involving twenty-eight patients recruited from the cardiomyopathy clinic at Cairo University Children’s Hospital. Inclusion criteria specified that participants must be of both sexes, aged between five and sixteen years (to be able to describe the extent of manifestations of heart failure accurately), diagnosed with dilated cardiomyopathy, and regularly monitored in our clinic. Exclusion criteria included other types of cardiomyopathy, the presence of congenital heart disease or systemic heart disease, and any overt causes of dyssynchrony, such as left bundle branch block (LBBB), right bundle branch block (RBBB), and atrioventricular block. Additionally, patients classified as NYHA class I and class IV were excluded to minimize bias, given the limited number of patients in these categories.

To establish a comparative baseline for advanced echocardiographic parameters, thirty age- and sex-matched controls from the outpatient clinic were also enrolled in the study.

### Study methods

#### History taking and examination extracted from patients’ files

Items collected were body surface area, age at the onset of heart failure manifestations, age at the time of examination (with subsequent calculation of the estimated duration of the illness), and sex, number of heart failure hospitalizations in the last year, last level of NT-Pro-BNP (N Terminal Pro-Brain natriuretic Peptide), serum creatinine and anti-failure medications used.

*-A modified more objective NYHA classification (Adapted from Amdani and Tavares* [6, 7] *studies)* was assessed for each patient, as follows:

**Table:** 

Feature	None	Mild	Moderate	Severe
Heart Failure Class				
NYHA (age ≥ 5 y)/Ross (age < 5 y)	I	II	III	IV
Manifestations	I can do Physical activities without getting tired	I feel tired when I do physical activities, but I do not stop doing them	I feel tired and need to interrupt my physical activities	I am very tied and have shortness of breath so I need to stay seated without doing any physical activity
**Heart Failure Hospitalizations in the Past Year**	None	1	2	≥ 2

As mentioned earlier, Class IV and I patients were excluded from the study recruitment process.

#### Advanced assessment of left ventricular functions and intraventricular flow analysis [8, 9]

Echocardiographic examinations were conducted using the GE E95 machine, with analyses performed where applicable utilizing the Echopac workstation. The following procedures were used:

#### Tissue doppler imaging

##### Assessment of LV E/E’ ratio

The average basal septal and mitral annular velocities were calculated and served as the denominator for the LV E/E’ ratio, while the peak early mitral inflow velocity was used as the numerator.

#### Two-Dimensional (2D) speckle tracking

- Acquisition: 3, 4, and 2-chamber views of the left ventricle were obtained during ECG gating, with a frame rate exceeding 60 frames per second.
- Post-Systolic Strain Index (PSI): The relevant parameter recorded from the analysis was the PSI, calculated using the formula: PSI= (Maximal Strain-Peak Systolic Strain)/Maximal strain*100 [10].

#### Three-Dimensional (3D) speckle tracking echocardiography

- Acquisition: Full-volume acquisition of the left ventricle was completed in a single beat, with simultaneous ECG recording using 3D 5 or 3 MHz probes.
- Software Analysis: A step-by-step software-guided analysis was performed to detect the endocardial border of the left ventricle during both diastole and systole. The functional imaging software automatically calculated the left ventricular end-diastolic volume (EDV), which was indexed to body surface area (BSA), as well as global longitudinal strain (GLS) and global area strain (GAS).

#### Blood speckle tracking study

- Color Doppler Acquisition: A 6 MHz probe was utilized to obtain a color Doppler apical four-chamber view, with the color sector covering the entire left ventricular cavity.
- Visualization of Vortex Formation: Prior to loop recording, the “show particles” mode was activated. The loop was then replayed, demonstrating vortex formation within the left ventricle. Using ECG synchronization, we could determine whether the left ventricular vortex formation occurred during systole or diastole.

### Statistical analysis

#### Sample size calculation

A power analysis was conducted to determine the minimum sample size required to detect a clinically meaningful difference in the timing of left ventricular vortex formation between children with idiopathic dilated cardiomyopathy (DCM) and healthy controls, as assessed by cardiac MRI. Based on data from Kheradvar et al. [11] indicating that approximately 60% of DCM patients exhibit late diastolic and/or systolic left ventricular vortex compared to 0% in normal children, a sample size calculation was performed using PS Power and Sample Size Calculations software (version 3.0.11). Assuming an α-error level of 0.05, a power of 80%, and a 1:1 case-control ratio, the analysis determined that a minimum of 27 children per group was needed to detect a 30% difference in the proportion of late diastolic and/or systolic vortex using a two-sided Fisher’s exact test.

#### Data analysis

Data analysis was performed using MedCalc software (trial version). Categorical variables are presented as numbers and percentages. Continuous data were assessed for normality using the Shapiro-Wilk test. Normally distributed continuous data are presented as mean ± standard deviation and compared using independent samples t-tests. Non-normally distributed continuous data are presented as median and interquartile range (IQR) and compared using Mann-Whitney U tests. Categorical data were compared using Chi-square tests or Fisher’s exact tests, as appropriate. *Where assumptions for Chi-square tests were not met*,* Fisher’s exact test was used.*

Independent variables that showed significant differences between patient subgroups were included in the model. Receiver operating characteristic (ROC) analysis was used to assess the diagnostic accuracy of independent variables significantly different between patient subgroups in predicting worse manifestations (NYHA class III). *Optimal cut-off values for continuous variables were determined based on Youden’s J statistics (maximizing the sum of sensitivity and specificity).* All statistical tests were two-sided, and a *p*-value of < 0.05 was considered statistically significant.

## Results

Table 1 summarizes the demographic characteristics of the study participants, comparing cases (*n* = 28) and controls (*n* = 30). The mean age at the time of the study was 7.9 ± 2 years in cases and 9 ± 2 years in the controls (*p* = 0.06). The mean BSA was 0.79 ± 0.1 m² in cases and 0.87 ± 0.1 m² in controls (*p* = 0.1). The majority of both groups were male, with 68% of cases and 70% of controls being male (*p* = 0.8).

**Table 1:** Demographic data in cases vs. controls

		Cases(*n*=28)	Controls (*n*=30)	*P*-Value
Age at time of the diagnosis (years) (mean ±SD)		7.9±2	9±2	0.06
BSA (m^2^) (%) (mean ±SD)		0.79±0.1	0.87±0.1	0.1
Sex *n* (%)	Male	19 (68%)	21 (70%)	0.8
	Female	9 (32%)	9 (30%)	

*Abbreviations*: *BSA* Body surface area

Significant differences were observed in echocardiographic parameters (Table 2) between cases and controls. Global longitudinal strain (GLS) was significantly lower in cases (11.6 ± 2%) compared to controls (24 ± 2%) (*p* < 0.001). Similarly, post-systolic strain index was higher in cases (3.9 ± 1.6) than in controls (1.1 ± 0.6) (p < 0.001). Global area strain (GAS) and left ventricular ejection fraction (LVEF) were also significantly reduced in cases (16 ± 5% and 27 ± 5%, respectively) compared to controls (38 ± 3% and 71 ± 3%, respectively) (*p* < 0.001). Left ventricular sphericity index (LV SPI) was higher in cases (0.5 ± 0.1) compared to controls (0.3 ± 0.02) (*p* < 0.001). The LV E/E’ ratio was also significantly higher in cases (10 ± 1) compared to controls (4 ± 1) (*p* < 0.001). Diastolic vortex was less frequent in cases (46%) compared to controls (100%) (*p* < 0.001), while systolic vortex was only present in cases (54%) and absent in controls (0%) (*p* < 0.001).

**Table 2:** Echocardiographic data in cases vs. controls

		Cases (*n*=28)	Controls (*n*=30)	*P*-Value
GLS (%) (mean±SD)		11.6±2	24±2	<0.001
Post-systolic strain index (%) (median (IQR))		4 (2.5-5)	1 (1-2)	<0.001
GAS (%) (mean±SD)		16±5	38±3	<0.001
LVEDVI (mL/m^2^) (%) (mean±SD)		88±19	70±4	<0.001
LVEF (%) (mean±SD)		27±5	71±3	<0.001
LV SPI (mean±SD)		0.5±0.1	0.3±0.02	<0.001
LV E/E’ (mean±SD)		10±1	4±1	<0.001
LV vortex *n* (%)	Diastolic	13 (46%)	30 (100%)	<0.001
	Systolic	15 (54%)	0 (0%)	

*Abbreviations*: *E/E’* Left ventricle diastolic index: ratio of early transmitral flow to the average early diastolic basal septal and mitral annular velocities, *EDVI* End-diastolic volume index, *EF* Ejection Fraction, *GAS* global area strain, *GLS* Global longitudinal strain, *IQR* Interquartile range, *LV* Left ventricle, *SPI* sphericity index

Table 3 presents the demographic and disease-related data for subgroups of cases categorized by NYHA class (II and III). The median age at onset of manifestations was 4 months (IQR 2.2–24) in NYHA class II and 3 months (IQR 1–4) in NYHA class III (*p* = 0.01). The mean age at the time of examination was 7 ± 1 years in NYHA class II and 8 ± 2 years in NYHA class III (*p* = 0.025). A small percentage of patients had a family history of similar conditions, with 7% in NYHA class II and 15% in NYHA class III (*p* = 0.4). The median duration of manifestations was longer in NYHA Class III subgroup being 8 ± 2 years compared to 5 ± 2 years in NYHA class II (*p* = 0.03).

**Table 3:** Demographic and disease data in subgroups of cases

			NYHA class II (*n*=15)	NYHA class III (*n*=13)	*P*-value
Age at onset of manifestations (months) median (IQR)			4 (2.2-24)	3(1-4)	0.01
Age at the Time of examination (years) (mean±SD)			7±1	8±2	0.025
Family History of Similar Conditions *n*(%)			1 (7)	2(15)	0.4
Duration of manifestations (years) median (mean±SD)			5±2	8±2	0.03
Sex *n* (%)	Male		10 (67%)	9 (69%)	0.89
	Female		5 (33%)	4 (31 %)	
BSA (m^2^) (mean±SD)			0.89 (0.67-0.94) 0.8±0.1	0.7(0.6-0.87) 0.7±0.2	0.5
HR (mean±SD)			103±9	103±11	0.9
NT-Pro-BNP (pg/mL) median (IQR)			510(422-772)	617 (557-840)	0.1
Duration between the timing of Pro-BNP testing and the study time (months) (mean±SD)			3.2±1.1	3.8±1.2	0.1
Serum Creatinine (mmol/L) (mean±SD)			68±14	62±15	0.2
Medications *n* (%)	ACEi/ARB	No	3 (20)	4 (31)	0.5
		Yes	12(80)	9 (69)	
	Sacubitril-Valsartan	No	12 (80)	9 (69)	0.1
		Yes	3 (20)	4 (31)	
	Furosemide	No	0(0)	0 (0)	1
		Yes	15 (!00)	13 (100)	
	Thiazide	No	14 (93)	10 (77)	0.2
		Yes	1 (7)	3 (23)	
	Spironolactone	No	1 (7)	1 (8)	0.9
		Yes	14 (93)	12 (92)	
	Digoxin	No	14 (93)	11 (85)	0.4
		Yes	1 (7)	2(15)	
	Ivabradine	No	8 (53)	8 (61.5)	0.6
		Yes	7 (47)	5 (38.5)	
	Carvedilol	No	13 (87)	13 (100)	0.17
		Yes	2 (13)	2 (13)	

*Abbreviations*: *BSA* Body surface are, *IQR* Interquartile range, *NYHA* New York Heart association, *NT-Pro-BNP* N terminal Pro-Brain natriuretic peptide

The majority of both groups were male, with 67% in NYHA class II and 69% in NYHA class III (*p* = 0.89). The mean BSA was 0.89 m² (IQR 0.67–0.94) in NYHA class II and 0.7 m² (IQR 0.6–0.87) in NYHA class III (*p* = 0.5). The mean heart rate was 103 ± 9 bpm in NYHA class II and 103 ± 11 bpm in NYHA class III (*p* = 0.9).

The median NT-Pro-BNP level was not significantly different between subgroups of cases being 510 pg/mL (IQR 422–772) in NYHA class II and 617 pg/mL (IQR 557–840) in NYHA class III (*p* = 0.1).

The mean serum creatinine level was 68 ± 14 mmol/L in NYHA class II and 62 ± 15 mmol/L in NYHA class III (*p* = 0.2).

None of the medications used were significantly prevalent in subgroups of cases despite slight differences between NYHA class II and III subgroups. The two overall most prevalent medications were ACEi/ARBs and Furosemide as follows: the use of ACEi/ARB was 80% in NYHA class II and 69% in NYHA class III (*p* = 0.5), the slightly lower use of ACEi/ARB in NYHA class III group might be attributed to the switching them to Sacubitril-Valsartan as it was implemented in 20% in NYHA class II and 31% in NYHA class III (*p* = 0.1). All patients in both groups were on Furosemide (*p* = 1) (Table 3).

Thiazides were more frequent in NYHA class III accounting for 23% in NYHA class III compared to 7% in NYHA class II (*p* = 0.2), possibly to overcome the phenomenon of diuretic resistance encountered with prolonged. The use of Spironolactone was 93% in NYHA class II and 92% in NYHA class III (*p* = 0.9). The use of Digoxin was 7% in NYHA class II and 15% in NYHA class III (*p* = 0.4). The use of Ivabradine was 47% in NYHA class II and 38.5% in NYHA class III (*p* = 0.6). The use of Carvedilol was 13% in NYHA class II and 0% in NYHA class III (*p* = 0.17).

When comparing subgroups of cases based on NYHA functional class, post-systolic strain index was significantly higher in NYHA class III (5.3 ± 0.7) compared to NYHA class II (2.7 ± 0.9)(*p* < 0.001). Diastolic vortex was more frequent in NYHA class II (67%) compared to NYHA class III (23%) (*p* = 0.02), while other advanced echocardiographic parameters failed to differentiated Class II and III NYHA subgroups (Table 4).

**Table 4:** Echocardiographic data in subgroups of cases

		NYHA class II(*n*=15)	NYHA class III(*n*=13)	*P*-Value
GLS (%) (mean±SD)		12±2	11±1	0.2
Post-systolic strain index(%) (mean±SD)		2.7±0.9	5.3±0.7	<0.001
GAS (%) (mean±SD)		16±5	15±5	0.7
LVEDVI (mL/m2) (%)(mean±SD)		87±20	89±18	0.6
LVEF (%)(mean±SD)		28±5	27±6	0.4
LV SPI (ratio) (mean±SD)		0.5±0.1	0.5±0.1	0.7
LV E/E’ (ratio) (mean±SD)		9±1	10±2	0.13
LV Vortex *n* (%)	Diastolic	10 (67%)	3 (23%)	0.02
	Systolic	5 (33%)	10 (77%)	

*Abbreviations*: *E/E’* Left ventricle diastolic index: ratio of early transmitral flow to the average early diastolic basal septal and mitral annular velocities, *EDVI* End-diastolic volume index, *EF* Ejection Fraction, *GAS* global area strain, *GLS* Global longitudinal strain, *IQR* Interquartile range, *LV* Left ventricle, *SPI* sphericity index

**Fig. 2:**
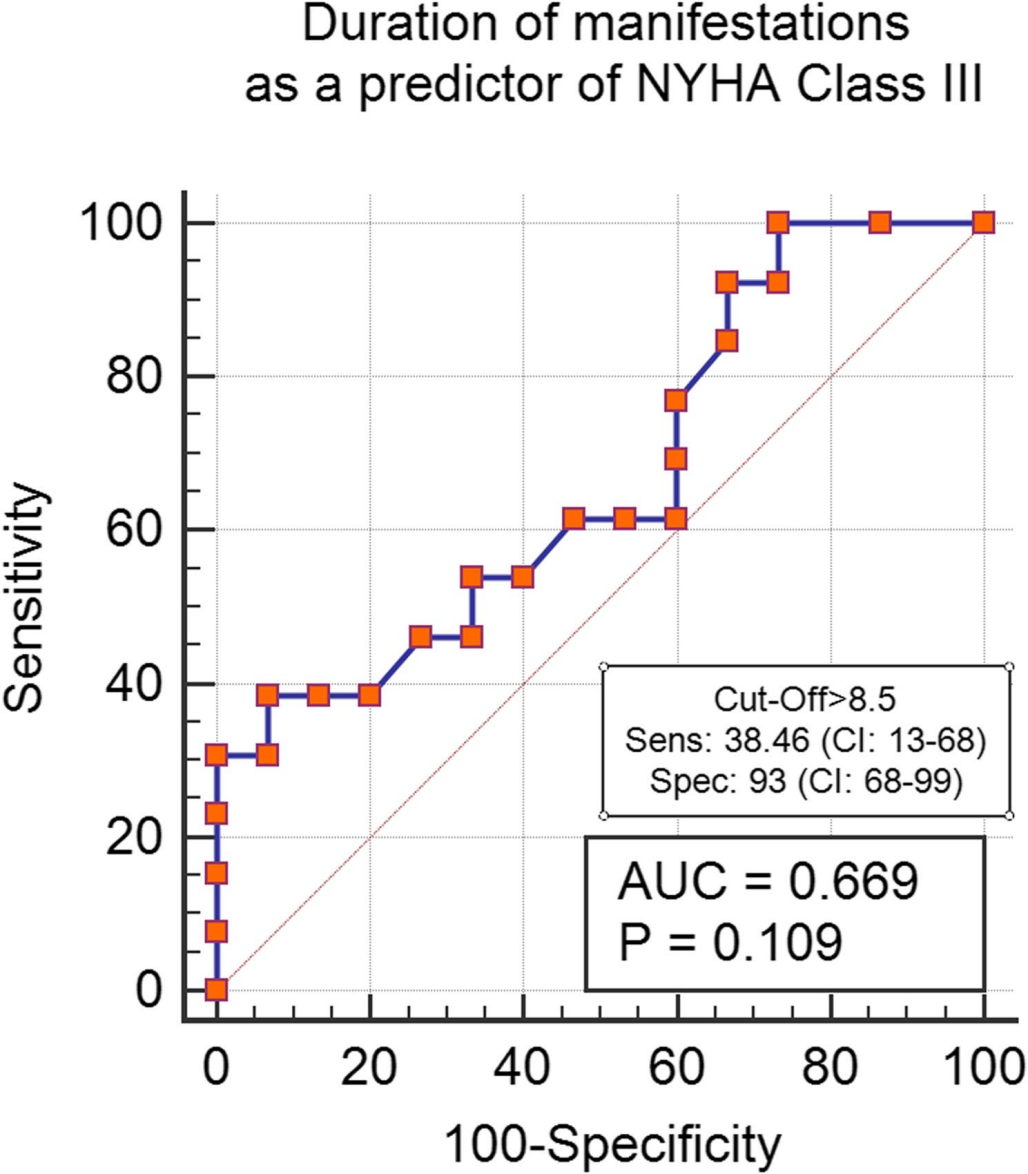
Post-systolic strain index as a predictor of NYHA Class III. Abbreviations: AUC: Area under the curve, NYHA: New York Heart association classification, Sens: sensitivity, Spec: Specificity

**Fig. 3:**
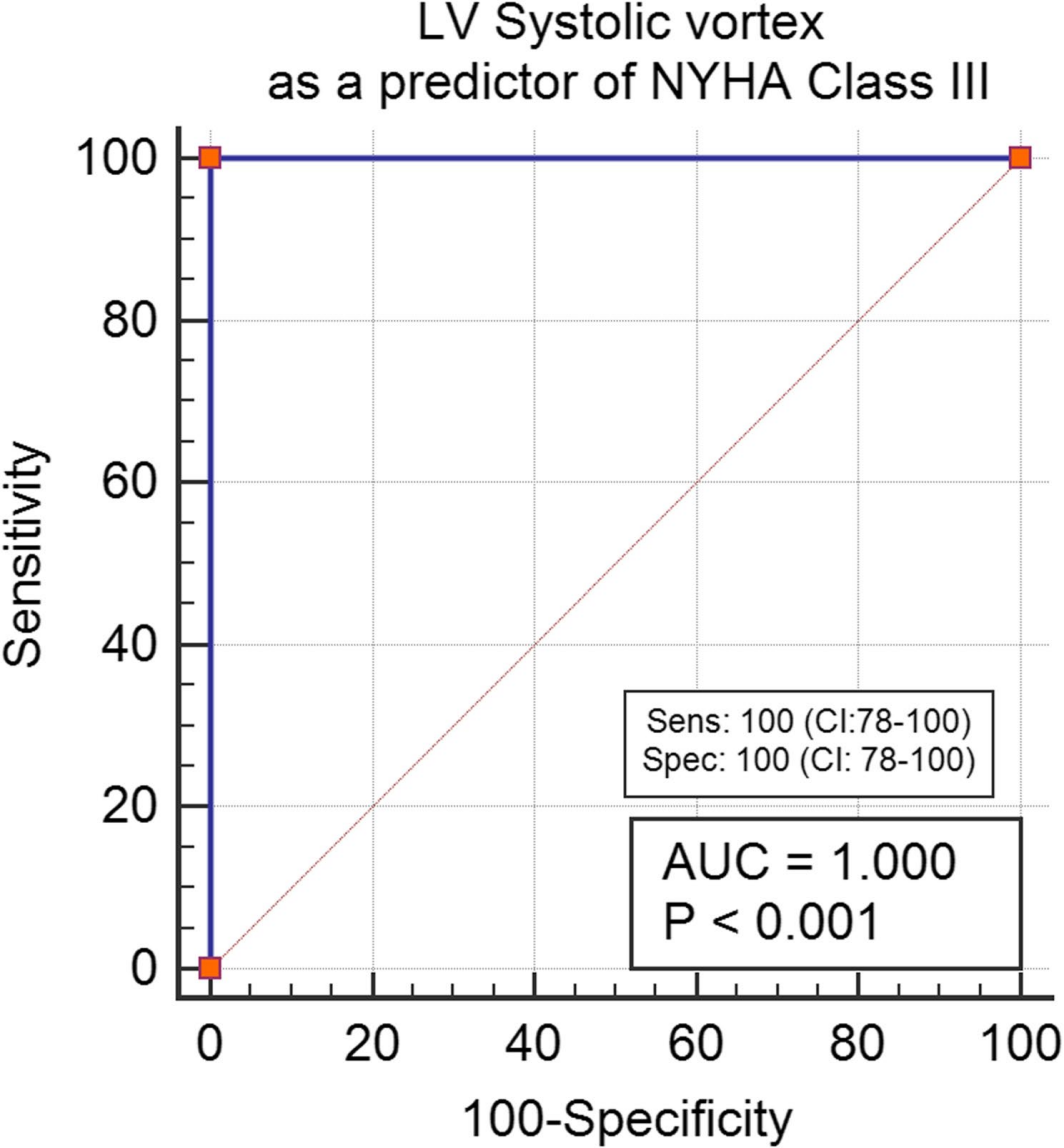
Duration of manifestations of heart failure as a predictor of NYHA Class III. Abbreviations: AUC: Area under the curve, NYHA: New York Heart association classification, Sens: sensitivity, Spec: Specificity

**Fig. 4:**
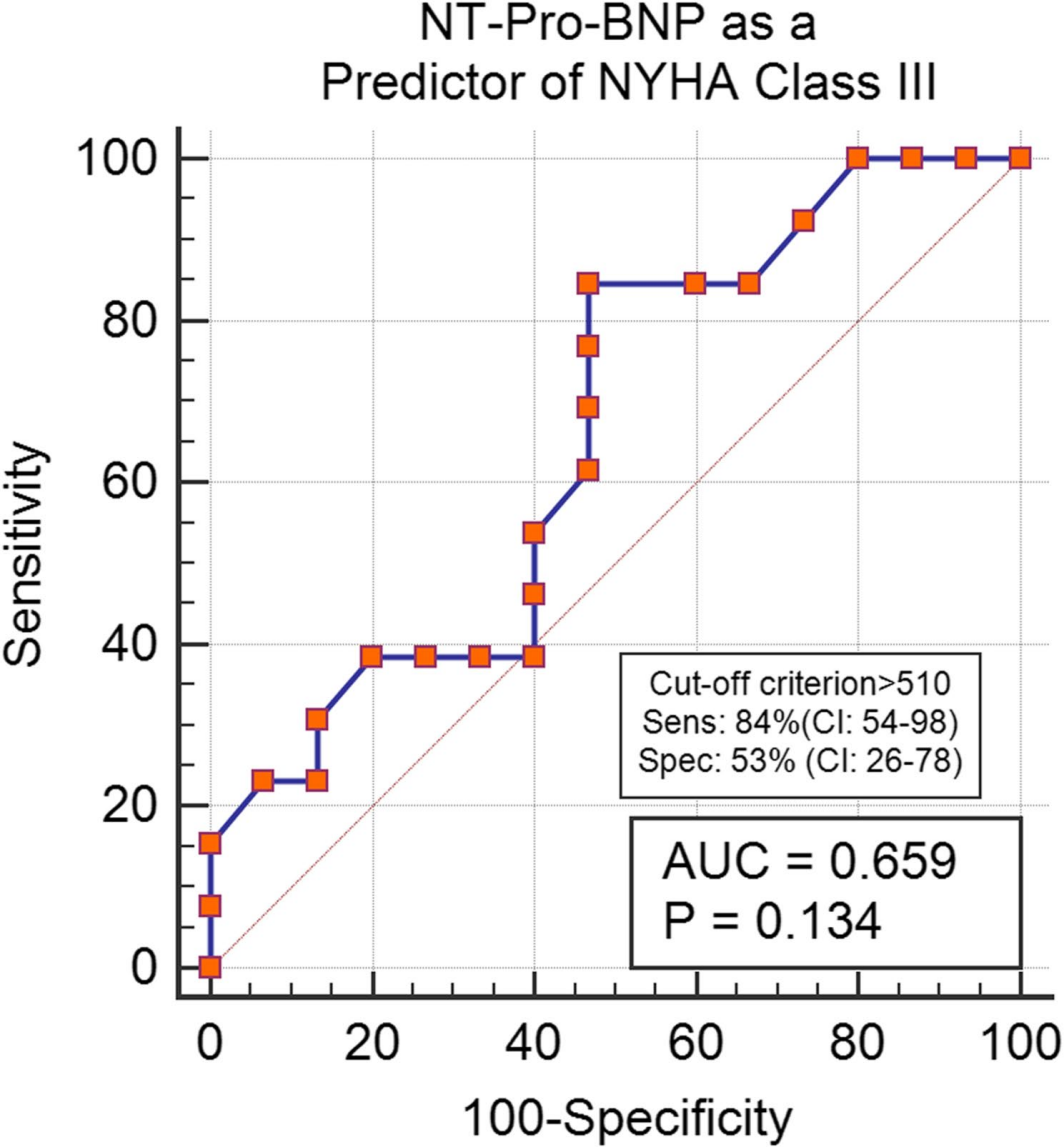
LV Systolic vortex formation as a predictor of NYHA Class III. Abbreviations: AUC: Area under the curve, NYHA: New York Heart association classification, Sens: sensitivity, Spec: Specificity

**Fig. 5:**
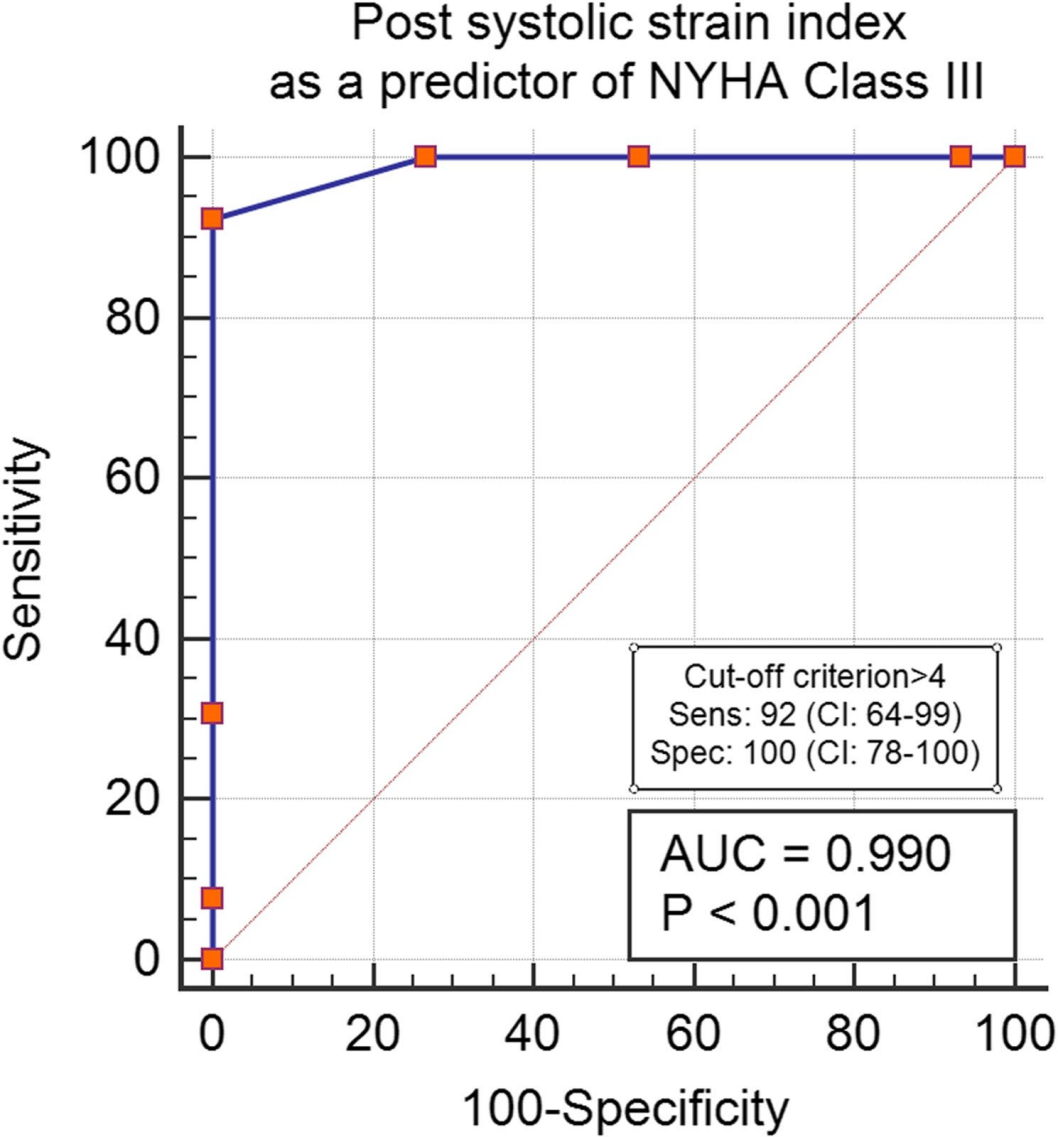
NT-Pro-BNP as a predictor of NYHA Class III. Abbreviations: AUC: Area under the curve, NYHA: New York Heart association classification, Sens: sensitivity, Spec: Specificity

The sensitivity and specificity of the manifestations’ duration, PSI, and systolic vortex were evaluated to predict the presence of more severe symptoms. A PSI greater than 4 demonstrated a high sensitivity of 92% (CI: 64–99) for predicting the NYHA III profile. In contrast, a duration of illness exceeding 8.5 years showed a lower sensitivity of 38% (*P* = 0.1) for indicating severe manifestations. Additionally, the visualization of a systolic vortex showed a sensitivity of 100% (CI: 78–100) for identifying the NYHA III subgroup. NT-Pro-BNP was 86% sensitive in prediction of NYHA Class III (CI: 54–98) (Refer to Figs. 2, 3, 4 and 5 for visual representation of these findings.)

**Fig. 6:**
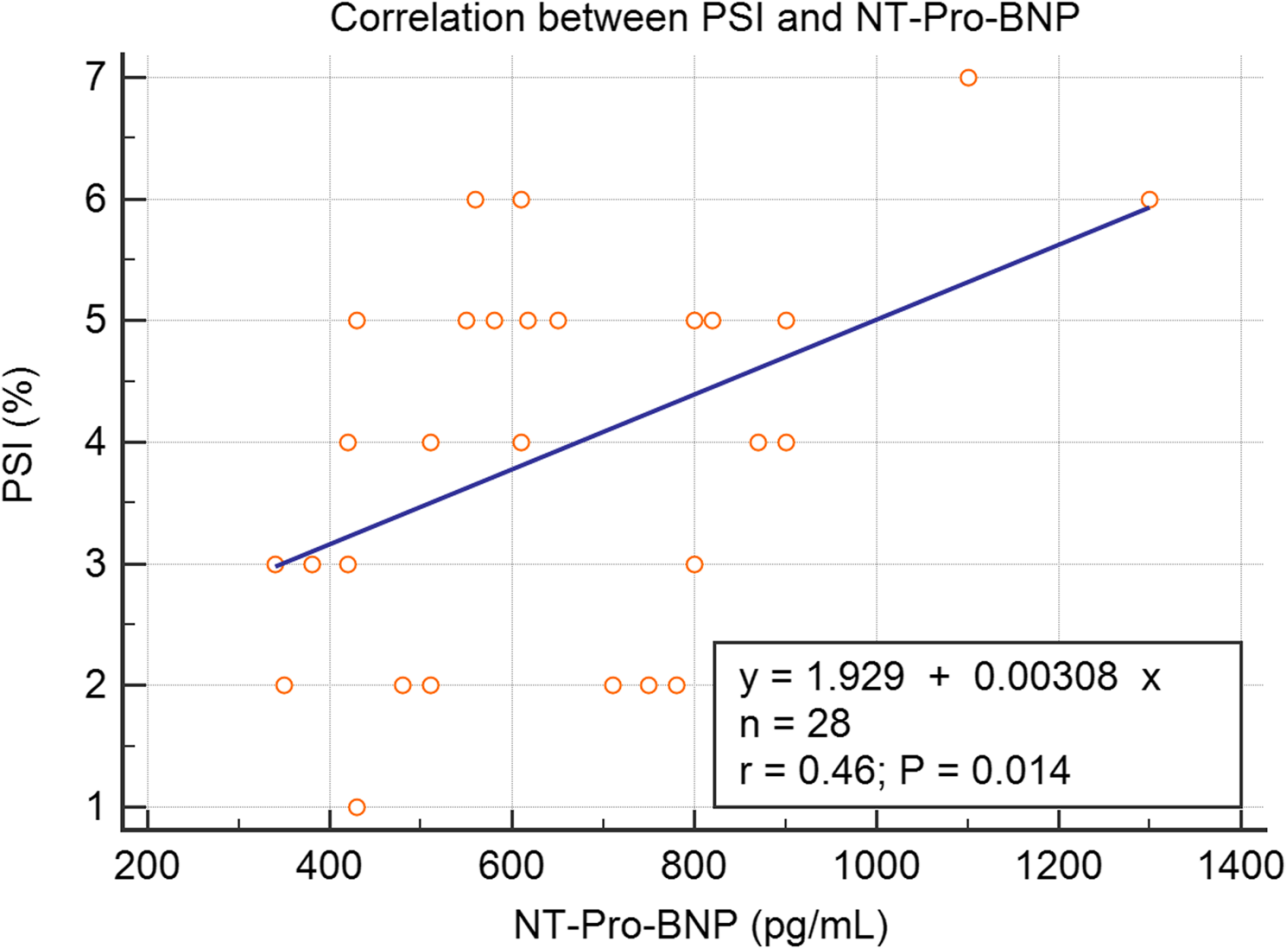
Title: Correlation between PSI and NT-Pro-BNP. Abbreviations: NT-Pro-BNP: N Terminal Pro-Brain Natriuretic Peptide, PSI: Post-systolic strain index, R: Correlation coefficient

There was a statistically significant positive correlation (Fig. 6) between PSI and NT-Pro-BNP with *r* = 0.6, *P* = 0.014.

As demonstrated by the scatterplot (Fig. 6), the correlation between PSI and NT-Pro-BNP, while statistically significant (*r* = 0.6, *p* = 0.014), is not strong. This suggests that while there is some association, these markers capture different aspects of cardiac dysfunction in our patient population. PSI may be more directly reflecting ventricular mechanics and dyssynchrony, while BNP, and this also can explain the loss of significance of BNP in multivariate analysis.

An inter-rater agreement (Kappa) analysis was conducted for the two most significant parameters of the study: post-systolic strain and left ventricular (LV) vortex. The Kappa index was found to be 0.64 for post-systolic strain and 0.90 for the LV vortex, indicating substantial to excellent agreement between raters for these measurements.

**Fig. 7:**
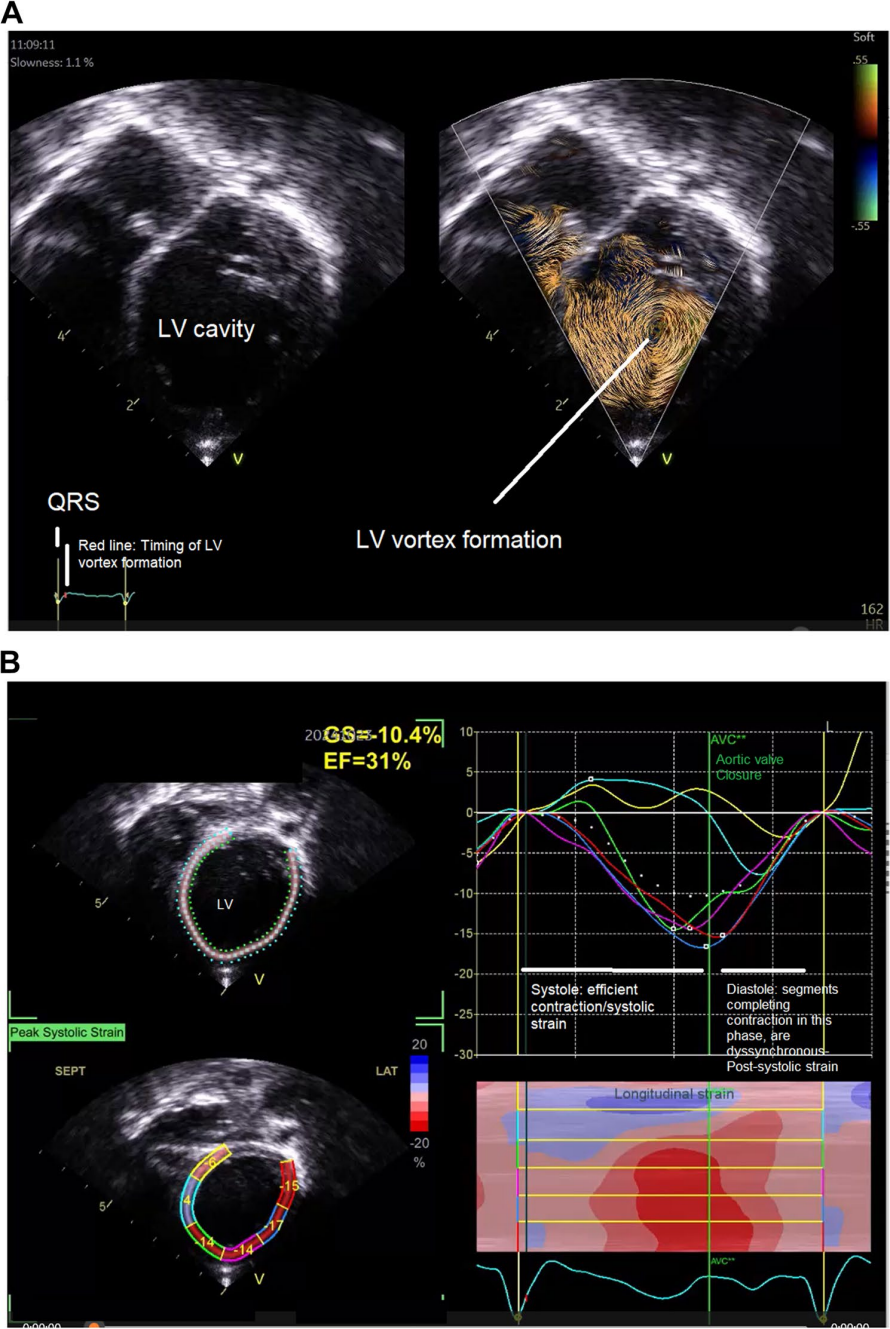
**A**: Systolic Vortex formation in a patient in this study **B**: 2D speckle Tracking showing post-systolic strain (after aortic valve closure in the same patient)

Figure 7A and B are representative of Vortex formation and Post-systolic strain in one of the patients included in the study.

## Discussion

Heart failure continues to be recognized as a complex clinical syndrome, despite the development of various robust echocardiographic parameters aimed at assessing cardiac function. There remains considerable uncertainty regarding the ability of these echocardiographic metrics to reliably predict adverse outcomes and accurately correlate with patients’ symptomatology. In this context, ejection fraction (EF) has drawn comparisons to serum creatinine levels in chronic kidney disease (CKD); both measures can be seen as inadequate proxies for the underlying severity of their respective conditions. Just as serum creatinine does not necessarily reflect the full spectrum of kidney dysfunction or the urgency for dialysis, ejection fraction may fail to capture the intricacies of heart failure’s clinical impact, overlooking the nuances of patient experience, functional capacity, and prognostic trajectory. This perspective highlights the need for a more holistic assessment approach, integrating multiple clinical, echocardiographic, and patient-reported factors to better understand and manage heart failure, ultimately improving care outcomes and patient quality of life [12]. This report aimed to clarify whether any echocardiographic parameters could effectively correlate with clinical manifestations and thereby serve as reliable prognostic indicators for heart failure cases. Notably, our study may be one of the first to explore the application of qualitative intraventricular flow analysis for this purpose. Despite observing significant discrepancies in advanced functional parameters between cases and controls, our findings revealed that these measures were insufficient for differentiating subgroups of cases based on New York Heart Association (NYHA) class. This outcome partially contrasts with the findings of Hasselberg et al., which demonstrated a significant correlation between peak oxygen consumption (VO2) and Minnesota questionnaire scores with global longitudinal strain (GLS). The divergence between our results and theirs underscores the need for further investigation into the complexities of echocardiographic metrics in heart failure, as it suggests that while some parameters may show promise, additional research is necessary to identify robust predictors of symptom severity and prognostic stratification based on clinical classifications [13, 14].

On another note, Late Gadolinium Enhancement (LGE) by CMR is a powerful tool in cardiac evaluation, particularly concerning risk stratification in patients with heart failure, independent of left ventricular ejection fraction (EF) or the presence of inducible ventricular tachycardia (VT). Research indicates that a myocardial LGE burden exceeding 5% is linked to a substantial 20% annual risk of death or significant arrhythmic events. In contrast, patients without any LGE show a markedly lower annual risk of around 3%. This stark difference in outcomes underscores the critical nature of LGE assessment in clinical practice. Furthermore, the specific locations, patterns, and extent of LGE can significantly influence prognostic outcomes. For example, LGE located in the lateral wall of the left ventricle, particularly when transmural, has been associated with a poor response to cardiac resynchronization therapy (CRT), highlighting the importance of tailoring treatment strategies based on imaging findings [3].

The absence of LGE, on the other hand, not only correlates with a favourable prognosis but also indicates a greater likelihood of reverse remodelling in response to optimized medical therapy. This signifies that patients without LGE tend to exhibit more favourable cardiac function improvements with appropriate medical management. Recent findings, particularly from studies like the DANISH trial, emphasize the growing importance of LGE assessment in patients with dilated cardiomyopathy (DCM). Such insights facilitate more individualized patient care, allowing clinicians to better predict disease progression and tailor therapeutic approaches. As understanding of LGE continues to evolve, it is becoming increasingly clear that LGE imaging is an essential component of comprehensive heart failure evaluation, offering critical prognostic information that can enhance clinical decision-making and ultimately improve patient outcomes [15, 16].

Dyssynchrony is widely recognized as a critical predictor of adverse outcomes, significantly contributing to impaired cardiac output in heart failure patients. Emerging evidence suggests that various forms of dilated cardiomyopathy (DCM) may initially manifest as a dyssynchronopathy, characterized by the abnormal timing of ventricular wall contractions. This disordered contraction not only affects the efficiency of the heart’s pumping action but also precipitates a cascade of progressive ventricular dysfunction. The disruption in synchronized contraction patterns can lead to suboptimal filling and emptying of the ventricles, exacerbating heart failure symptoms and increasing the risk of further cardiac complications [4, 10, 17, 18].

Altered flow patterns, particularly the presence of a systolic vortex, can be both a consequence and a driver of dyssynchrony and impaired cardiac output. As the heart’s normal contraction sequence is disrupted—either due to intrinsic myocardial abnormalities or external factors such as conduction delays—flow dynamics within the left ventricle become aberrant. The formation of a systolic vortex, characterized by swirling blood flow, often arises in response to these mechanical irregularities, reflecting the underlying dyssynchrony that affects effective ventricular filling and emptying. Conversely, this altered flow can contribute to further impairment in cardiac performance by leading to inefficient valve opening, incomplete chamber emptying, and increased energy losses during systole. Thus, the interplay between altered flow and dyssynchrony creates a vicious cycle: while the systolic vortex is indicative of existing dysfunction, it simultaneously exacerbates the heart’s inefficiency, complicating the clinical landscape for patients with heart failure and suggesting that addressing flow dynamics might offer a novel avenue for therapeutic interventions aimed at restoring synchronization and enhancing cardiac output [19, 20].

In this study, measures of dyssynchrony, specifically the post-systolic strain index (PSI) and the presence of a systolic vortex, demonstrated significant variations across different functional groups, providing a more accurate prediction of adverse manifestations than any other echocardiographic variable analyzed. These findings reinforce the understanding of dyssynchrony’s critical role in compromising cardiac function and exacerbating the symptoms of heart failure. Additionally, the assessment of intracardiac flow abnormalities shows promise in correlating with patients’ functional status. However, it is important to note that these measures remain qualitative and are dependent on the operator’s skill and interpretation.

The clinical utility of the aforementioned parameters is particularly significant in the detection of refractory heart failure despite optimized therapy. Many patients with heart failure remain symptomatic even after reaching target doses of medications, exhibiting comparable NT-Pro-BNP levels and ejection fractions to those who respond well to treatment [21–23]. Early identification of dyssynchrony in this subset is crucial for improving clinical outcomes. Reversal of dyssynchrony can be achieved not only through pacing strategies but also via rate-control medications such as Ivabradine [24].

Furthermore, the role of vortical flow dynamics in pediatric cardiac disease remains underexplored, with very few studies investigating its significance. To date, no research has specifically focused on vortex behavior in pediatric heart failure or cardiomyopathy. Notably, a study by Marchese and colleagues observed a progressive shift of vortex formation from near the interventricular septum in patients with congenital heart disease [25]. They also identified a transition of vortex activity from early diastole to the late ventricular filling phase. Similarly, Hu et al. evaluated vortex volume using 4D flow CMR in healthy children compared to those with repaired Tetralogy of Fallot. Their findings demonstrated a gradual reduction in vortex volume following repair and a shift in vortex timing from early diastole to systole. While these studies address different patient populations and pathologies, they collectively highlight that vortex dynamics—particularly timing and volume—are influenced by alterations in myocardial configuration and loading conditions, as seen in volume load lesions, repaired congenital defects, and idiopathic dilated cardiomyopathy in our study [26].

As we continue to refine these techniques and validate their prognostic value, a move toward more standardized and quantitative assessments may enhance our ability to improve patient management and outcomes in heart failure [19, 27].

## Conclusion

Intracardiac flow analysis is currently made available, using bedside echocardiography; their use and acquisition are seamless, and technically less demanding than speckle tracking echocardiography; there is still more to add in terms of analyses of the vortex size, [proximity to the septum and free wall and direct automated timing, however, it could point to the severity of heart failure in DCM patients, and went in agreement with post-systolic strain index in predicting the NYHA functional status of the patient. This can serve as a ground for more studies enrolling the same technology. In addition, dyssynchrony is intriguingly better for the prognostication of heart failure patients; however, no clinician can tell if it is the cause or the result of severe LV dysfunction.

## Limitations of the study

1. Limited Generalizability: The exclusion of patients in NYHA classes I and IV, along with those presenting with right or left bundle branch block, restricts the study’s applicability to a narrow range of heart failure severity. This, combined with a small sample size of only 28 patients, limits the statistical power and the ability to perform comprehensive multivariate analyses, potentially affecting the broader relevance of the findings.
2. Lack of Longitudinal Data: The cross-sectional design precludes any conclusions about the long-term prognostic value of PSI or vortex formation. Future longitudinal studies are essential to determine if these parameters can predict disease progression, hospitalization rates, or overall survival.
3. Subjectivity in Vortex Assessment: The visual categorization of vortex formation as “systolic” or “diastolic” lacks a formal assessment of interobserver variability and reproducibility. To enhance objectivity, future studies should incorporate quantitative measures of vortex characteristics, such as vortex intensity and vorticity indices, especially considering the operator-dependent nature of blood speckle tracking.

## Future recommendations

Future research should address the limitations of this study by expanding the patient population to include all NYHA functional classes and accounting for the presence of right or left bundle branch block to enhance generalizability. Longitudinal studies are needed to assess the prognostic value of PSI and vortex formation over time, tracking disease progression, hospitalization rates, and survival outcomes. To improve objectivity and reliability, future research should incorporate quantitative methods for assessing vortex formation, such as measuring vortex intensity and vorticity indices, and assess interobserver variability. Larger sample sizes are crucial to increase statistical power and enable robust multivariate analyses, allowing for more reliable conclusions. Furthermore, future studies should benchmark findings against cardiac magnetic resonance (CMR) imaging, a gold standard for cardiac assessment, to validate the accuracy and clinical relevance of PSI and vortex formation measurements. Finally, further research is needed to explore the clinical implications of PSI and vortex formation in heart failure, investigating their relationship to established markers of disease severity and prognosis, and assessing their potential utility in guiding treatment decisions. Future studies could validate the reproducibility of vortex timing across different operators and equipment settings.

## Supplementary Information

Supplementary Material 1.

## Data Availability

No datasets were generated or analysed during the current study.
