# Supplementary figures and images for: Blood speckle tracking to predict functional status in pediatric patients with dilated cardiomyopathy
Source: BMC Cardiovasc Disord. 2025 Jul 21;25:532. doi: 10.1186/s12872-025-04984-2 (PMC12278534; doi:10.1186/s12872-025-04984-2)

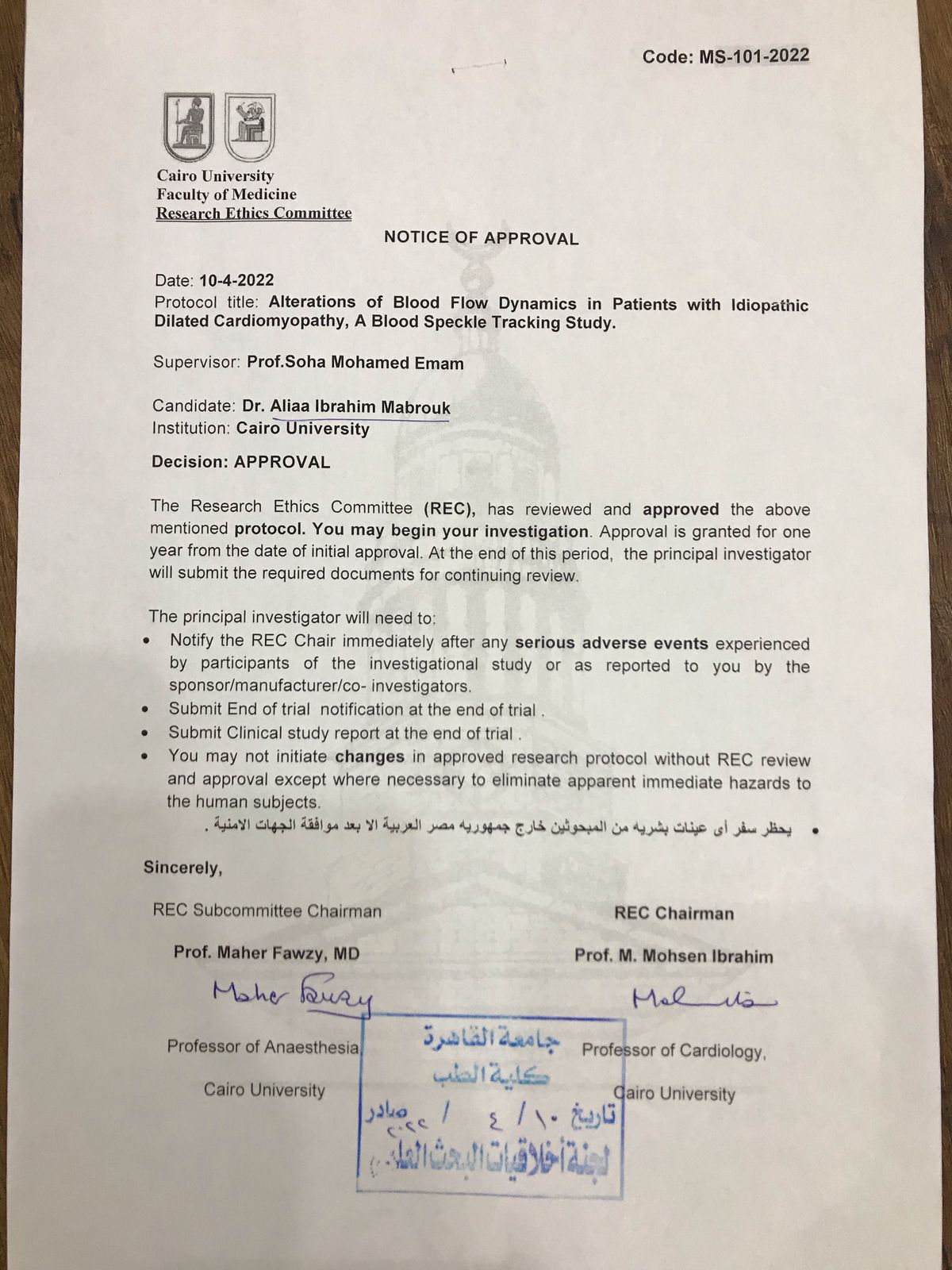

Supplement: Supplementary file 1 — Supplementary Material 1. [file 12872_2025_4984_MOESM1_ESM.jpg]
